# Efficacy and Safety of *Panax Notoginseng Saponins* (Xueshuantong) in Patients With Acute Ischemic Stroke (EXPECT) Trial: Rationale and Design

**DOI:** 10.3389/fphar.2021.648921

**Published:** 2021-04-22

**Authors:** Luda Feng, Fang Han, Li Zhou, Shengxian Wu, Yawei Du, Dandan Zhang, Chi Zhang, Ying Gao

**Affiliations:** ^1^ Institute for Brain Disorders, Beijing University of Chinese Medicine, Beijing, China; ^2^ Department of Neurology, Dongzhimen Hospital, Beijing University of Chinese Medicine, Beijing, China; ^3^ Beijing University of Chinese Medicine, Beijing, China; ^4^ Office of Academic Research, Beijing Hospital of Traditional Chinese Medicine, Capital Medical University, Beijing, China; ^5^ Office of State Drug Clinical Trial Institution, Dongzhimen Hospital, Beijing University of Chinese Medicine, Beijing, China; ^6^ Dongzhimen Hospital, Beijing University of Chinese Medicine, Beijing, China

**Keywords:** acute ischemic stroke, panax notoginseng saponins, xueshuantong, efficacy, safety, randomized controlled trial

## Abstract

**Background:** Although revascularization treatment is recommended as the first-line therapy for patients with non-minor acute ischemic stroke (AIS), it only benefits a minority of patients. Previous studies have reported the positive effects of *Panax notoginseng saponins* (PNS) (Xueshuantong lyophilized powder) on AIS, however, there have been no rigorous trials. This study aims to assess the efficacy and safety of PNS therapy for patients with AIS.

**Methods:** The Evaluation of Xueshuantong in Patients with acutE ischemiC sTroke (EXPECT) trial is a multicenter, randomized, placebo-controlled, double-blind study aiming to enroll 480 patients in China. Eligible patients with AIS within 72 h of symptom onset will randomly receive either PNS or PNS placebo for 10 days and subsequently be followed up to 90 days. The primary outcome will be a change in the National Institute of Health Stroke Scale (NIHSS) score from baseline to 10 post-randomization days. The secondary outcomes include early neurological improvement (proportion of patients with NIHSS score 0–1), and Patient-Reported Outcomes Scale for Stroke score at 10 post-randomization days, the proportion of patients with life independence (modified Rankin Scale score of 0–1), the proportion of patients with a favorable outcome (Barthel Index ≥90), and Stroke-Specific Quality of Life score at 90 days. Adverse events or clinically significant changes in vital signs and laboratory parameters, regardless of the severity, will be recorded during the trial to assess the safety of PNS.

**Conclusions:** To our knowledge, this study is the first double-blind trial to assess the efficacy and safety of PNS in patients with AIS. Findings of the EXPECT trial will be valuable in improving evidence regarding the clinical application of PNS therapy in patients with AIS ineligible for revascularization treatment in the reperfusion era.

## Introduction

Stroke is the second leading mortality cause worldwide and ranks first in China (Zhou et al., 2019; GBD 2019 Diseases and Injuries Collaborators, 2020). The high rates of stroke prevalence, incidence, and disability cause a significant economic burden (Rajsic et al., 2019; Wu et al., 2019a). Ischemic stroke is the most common stroke subtype, accounting for approximately 70% of all stroke cases (Wang et al., 2017). Currently, revascularization treatment within 24 h of symptom onset is recommended for saving the penumbra to improve functional outcomes in patients with non-minor acute ischemic stroke (AIS) (Powers et al., 2019). Since 1995, intravenous thrombolysis has been administered as the first-line therapy for AIS (NINDS, 1995). However, it benefits a limited number of patients given the narrow time-window, prevalent patient delay, imaging dependence, and risk of hemorrhagic transformation (Yaghi et al., 2017; Powers et al., 2019). Additionally, despite the extended time-window of endovascular thrombectomy, it has limited clinical application since it requires superior surgical skills, advanced catheter, rapid neuroimaging evaluation of the core infarction territory, and extensive economic resources (Report on Stroke Prevention and Treatment in China Writing Group, 2020). Patients with non-minor stroke presenting a National Institute of Health Stroke Scale (NIHSS) score higher or equal to four are likely to have unfavorable functional outcomes once they miss the critical treatment opportunity at the acute stage. Therefore, there is a substantial need to develop effective and safe therapies benefiting a large number of patients with AIS.

The pathophysiology of cerebral ischemic injury is a complex and dynamic process, during which, the temporal and spatial evolution of a rapid cascade of events including energy failure, excitotoxicity, oxidative and nitrative stress, and inflammatory response is associated with tissue damage following cerebral ischemia (Dirnagl et al., 1999; Lo et al., 2005; Chamorro et al., 2016). Among them, inflammatory injuries are triggered within minutes and last for weeks. Injured brain cells extensively produce pro-inflammatory cytokines, including interleukin-6 (IL-6) and tumor necrosis factor-alpha (TNF-α), which results in neuronal damage. Consequently, there is an increased expression of adhesion molecules, including intercellular adhesion molecule 1 (ICAM-1), on the endothelial cell surface, which increases endothelial cell permeability, and therefore exacerbates ischemic injury (Barone et al., 1997; Dirnagl et al., 1999). Additionally, the accumulation of inflammatory mediators leads to blood-brain barrier disruption during the early phase after stroke onset (Brea et al., 2009; Giraud et al., 2015). As a result, leukocytes infiltrate the injured brain region and aggravate blood-brain barrier disruption in turn by releasing pro-inflammatory cytokines and matrix metalloproteinases (Neumann et al., 2015). Apart from focal inflammation, the so-called global inflammation responses occur and persist throughout the entire brain, affecting patients’ clinical outcomes (Shi et al., 2019). Therefore, neuroinflammation is deemed as the potential treatment target (Jayaraj et al., 2019).

*Panax notoginseng saponins* (PNS) (Xueshuantong lyophilized powder) isolated from the roots and rhizomes of Panax notoginseng (Burkill) F.H.Chen consists of five main components: notoginsenoside R1, ginsenoside Rg1, ginsenoside Rd, ginsenoside Rb1, and ginsenoside Re (*see* Additional File 1 in Supplementary Material). The systematic pharmacokinetics of PNS indicates that the main circulating constituents are unchanged saponins, and intravenous PNS administration guarantees drug stability without inducing cytochrome P450 3A (Pintusophon et al., 2019; Zhang et al., 2020). PNS has been shown to exert strong anti-inflammatory effects against atherosclerosis-related cardiac-cerebral vascular disease (Wan et al., 2009; Wang et al., 2011). Both *in vitro* and *in vivo* studies have proved that PNS and notoginsenoside R1 significantly reduced the levels of IL-6, TNF-α, and ICAM-1 via microRNA downregulation, inhibiting NF-κB signaling pathway activation, and increasing the anti-inflammatory factor levels (Huang et al., 2015; Shi et al., 2017; Fu et al., 2018; Meng et al., 2019). Besides, PNS and ginsenoside Rb1 have been reported to attenuate ischemia-reperfusion-induced degradation of endothelial tight junctions by inhibiting matrix metalloproteinase-9 (MMP-9) expression and increasing the tissue inhibitor levels of metalloproteinase, which alleviates blood-brain barrier disruption (Chen et al., 2015; Wu et al., 2019b). Other studies have demonstrated the neuroprotective effects of PNS concerning antioxidant capacity (Zhang et al., 2019), anti-apoptosis (Chen et al., 2011), and endothelial cell protection (Hu et al., 2018).

PNS administration to patients with AIS within 72 h of symptom onset improves local brain perfusion and promotes the structural plasticity of white matter fibers (Gui et al., 2013; Ren et al., 2018). However, these findings were reported by small-scale, open-label, single-center studies, which limited the robustness of the conclusions. It remains unclear whether patients with non-minor stroke could benefit from PNS therapy. Therefore, there is a need for a large-scale, well-designed, randomized controlled trial with clinical endpoints to determine the effects of PNS on patients with AIS. We further hypothesize that short-term treatment with PNS for patients with AIS could effectively decrease the NIHSS score. We, therefore, designed the **E**valuation of **X**ueshuantong in **P**atients with acut**E** ischemi**C** s**T**roke (EXPECT) trial to assess the efficacy and safety of PNS in patients with AIS.

## Methods and Design

### Study Design

The EXPECT trial (Clinicaltrials.gov, NCT04415164) is a prospective, multicenter, randomized, placebo-controlled, double-blind study to test the hypothesis that PNS is superior to placebo in decreasing the NIHSS score of patients with AIS after 10 days. This trial protocol was approved by the institutional review board of Dongzhimen Hospital, Beijing University of Chinese Medicine, Beijing, China (No. DZMEC-JG-2019-51-01). We described this protocol according to the SPIRIT 2013 Statement (Chan et al., 2013) and the complete checklist is available (*see* Additional File 2 in Supplementary Material).

### Patient Selection

We will recruit patients diagnosed as AIS with an NIHSS score of 4–16 (a total score of upper and lower limbs ≥2 on motor deficits), who can be randomized within 72 h of symptom onset, which is defined based on the “last seen normal” principle. The age of recruited patients will be limited to 18–80 years. All patients or their legally authorized representatives will provide written informed consent before any study-specific procedure. Table 1 lists the detailed inclusion and exclusion criteria.

**TABLE 1:** Inclusion and exclusion criteria of the EXPECT trial.

**Inclusion criteria**
Acute ischemic stroke confirmed by head CT or MRI
Female or male patient aged ≥18 years and ≤80 years
Time from symptom onset to the randomization ≤72 h
4 ≤ NIHSS score ≤16 (total score of upper and lower limbs on motor deficits ≥2) at the randomization time
Signed informed consent
**Exclusion criteria**
Having already received thrombolysis or endovascular treatment before randomization
Secondary stroke caused by a tumor, traumatic brain injury, hematological disease, or other diseases with a confirmed diagnosis
Preceding mRS score ≥2
Other conditions that cause motor dysfunction (claudication, severe osteoarthrosis, rheumatoid arthritis, gouty arthritis, etc.).
Known severe liver or kidney dysfunction
Known allergies for ingredients in the investigational product, allergy history for food or medicine
Known medical condition likely to limit survival to less than 3 months
Known massive cerebral infarction combined with disturbance of consciousness (1a ≥2 in the NIHSS), dementia, mental impairment, or unsuitability for participation as judged by the investigators
Pregnancy or breastfeeding
Having participated in another clinical trial within 3 months before randomization

NIHSS, National Institute of Health Stroke Scale; mRS, modified Rankin Scale.

### Randomization, Allocation, and Blinding

**FIGURE 1:**
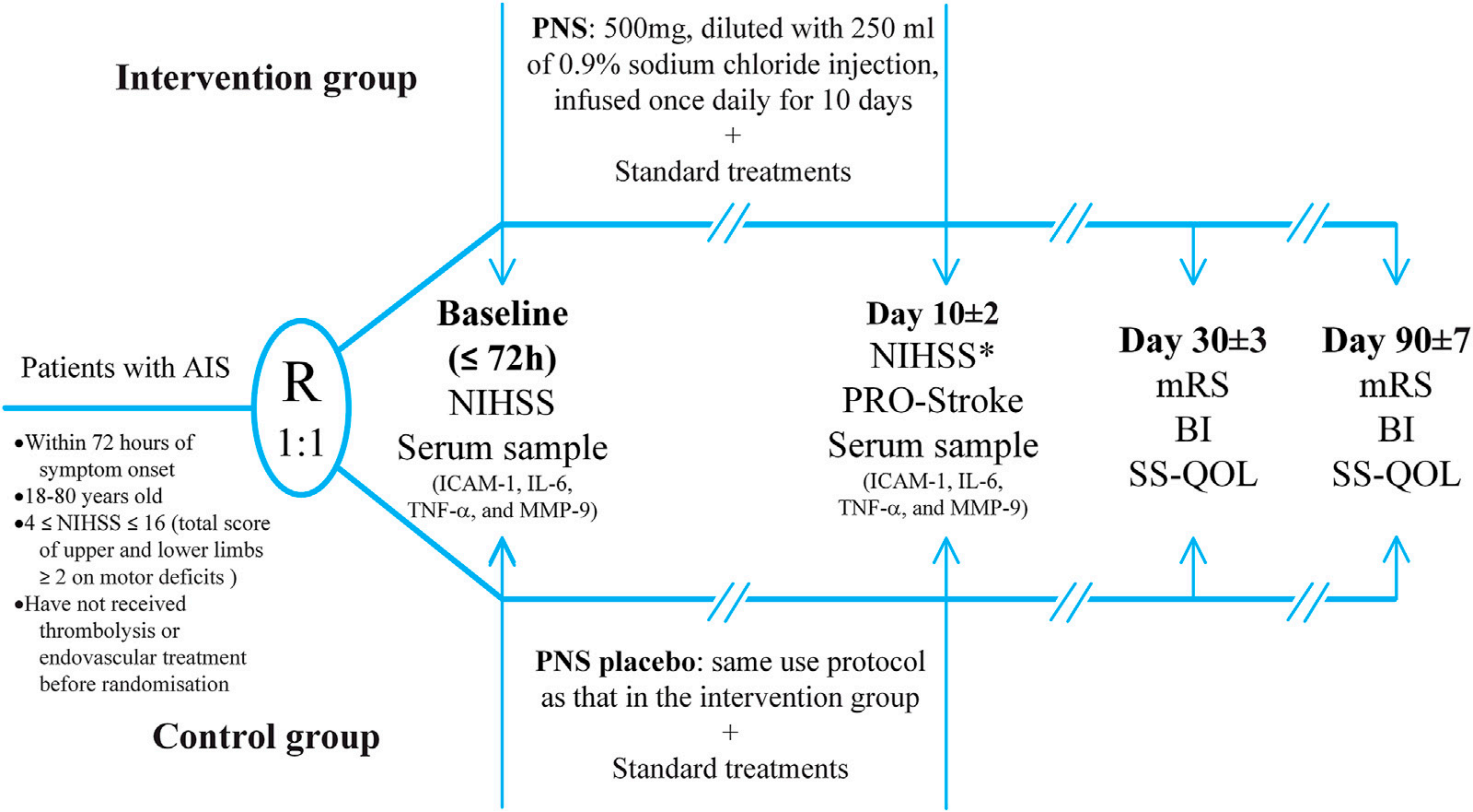
The flowchart of the EXPECT trial.

The investigators will randomize 480 eligible patients and assign them to the intervention and control groups at a 1:1 ratio using block randomization with stratification according to medical centers. The randomization schedule will be generated by an independent statistician using SAS software version 9.4 (SAS Institute Inc.) and kept in sealed, sequentially numbered, opaque envelopes. Investigational medicine blinding will be completed at a pharmaceutical factory based on the randomization schedule and sent to medical centers along with the emergency envelopes. The block size will be closed to ensure concealment throughout the entire trial period. All investigators, participants, caregivers, and data analysts will be blinded to the treatment assignments throughout the trial until the blind codes are unconcealed. Investigators can request emergency unblinding in case of serious adverse events (SAEs) suspected to be associated with investigational medicine. Figure 1 presents a flowchart of the EXPECT trial.

### Treatment

Eligible patients will be assigned to the intervention and control groups. The intervention group will receive daily single infusions of 500 mg PNS diluted with 250 ml of 0.9% sodium chloride injection for 10 days. The control group will receive a PNS placebo using the aforementioned protocol. The PNS and PNS placebo will be manufactured and supplied by Guangxi Wuzhou Pharmaceutical (Group) Co., Ltd. with identical appearance, color, and flavor. All patients will receive current guideline-recommended standard treatments, including the basic control of risk factors and anti-platelets for AIS (Powers et al., 2019). Figure 1 presents the treatment assignments. Edaravone and butylphthalide administration will be strictly prohibited during the treatment period. Investigational medicine will be discontinued in case of SAE occurrence, study withdrawal request from the patients or their legally authorized representatives, or poor compliance or non-adherence to the prescribed interventions. We will faithfully record the reasons for discontinuing interventions.

### Study Settings and Recruitment Strategies

**FIGURE 2:**
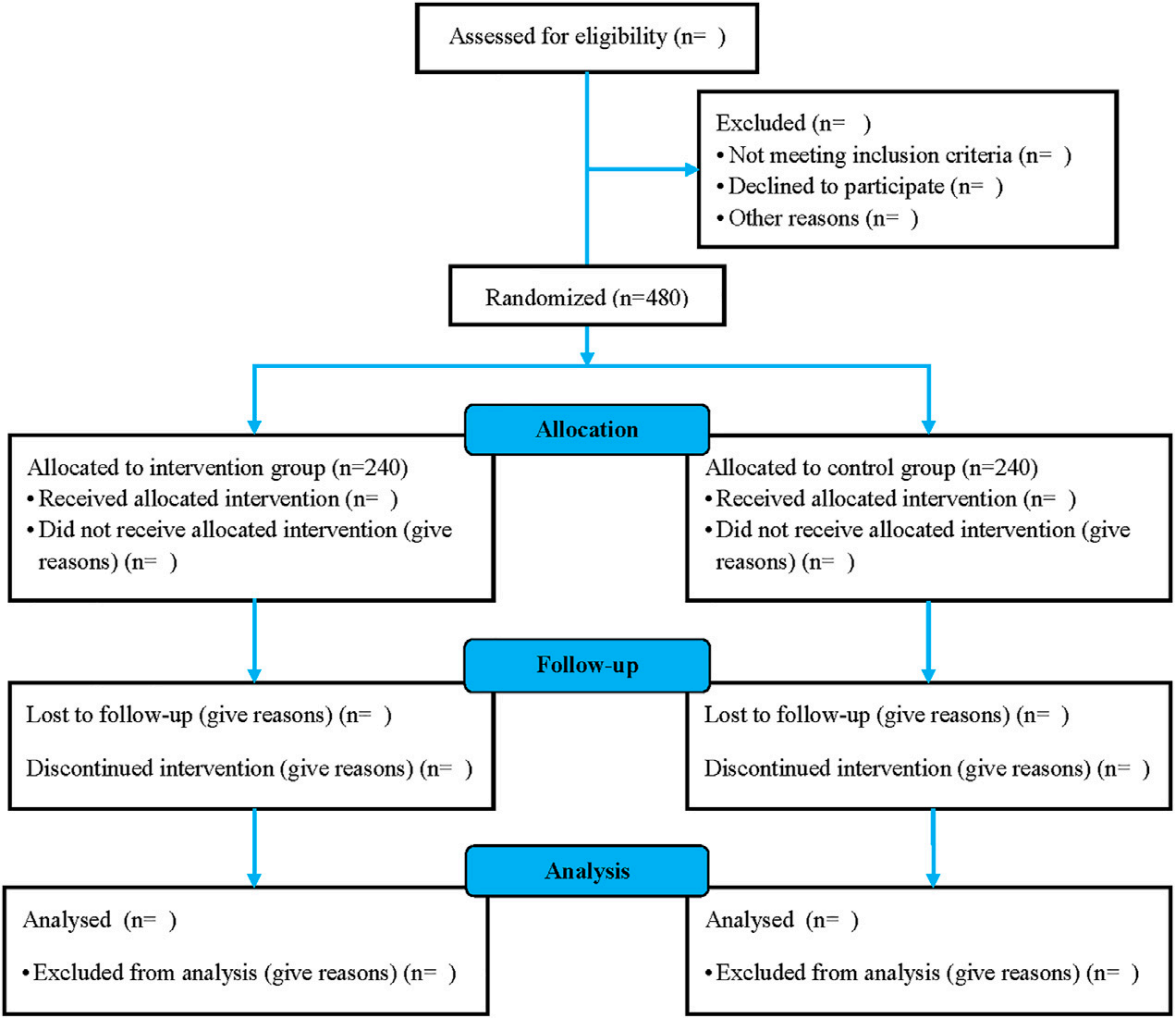
The schematic diagram of the patient timeline.

Inpatients will be recruited from 11 tertiary hospitals throughout eight provinces in China. Additionally, poster advertisements will be placed in these medical centers to allow the patients to voluntarily contact investigators. Potential patients will be screened for eligibility based on the inclusion and exclusion criteria. Eligible patients will be informed regarding the risks and benefits of the study. Subsequently, patients or their legally authorized representatives will sign the informed consent form if they agree to participate in the study. Patient enrollment of the EXPECT trial began in September 2020. Until December 2020, 13 patients had been enrolled and the estimated primary completion will be October 2022. Figure 2 presents a schematic diagram of the patient timeline.

### Efficacy Outcomes

In this trial, the primary outcome is neurological deficit amelioration defined as a change in the NIHSS score from baseline to 10 post-randomization days. Secondary outcomes will be as follows: 1) the proportion of patients with early neurological improvement (NIHSS score 0–1) at 10 post-randomization days; 2) patients’ subjective feelings measured at 10 post-randomization days using the Patient-Reported Outcomes scale for Stroke (PRO-Stroke), which is a well-validated scale suitable in Chinese patients for assessing stroke and treatment effects on physical function, psychological change, social engagement, and treatment satisfaction (Wang et al., 2012a; Wang et al., 2012b; Wang et al., 2012c); 3) proportion of patients with life independence (90-days modified Rankin Scale [mRS] score ≤1); 4) proportion of patients with a favorable outcome (90 days BI score ≥90); and 5) patients’ quality of life measured using the Stroke-Specific Quality of Life (SS-QOL) at 90 days.

### Biological Outcomes

We will perform between-group comparisons of the changes in ICAM-1, IL-6, TNF-α, and MMP-9 levels from baseline to 10 post-randomization days.

### Safety Outcomes

The safety outcome will include any adverse events (AEs), SAEs, and clinically meaningful changes in vital signs or laboratory parameters during the trial period.

### Follow-Up Procedures

The EXPECT trial contains four visits including time at randomization (baseline), 10 ± 2 days after randomization, 30 ± 3 days, and 90 ± 7 days after stroke onset. At baseline, we will evaluate demographic characteristics, routine laboratory tests, non-contrast CT/MRI, vessel imaging (carotid artery ultrasound/transcranial Doppler imaging/MR angiography), electrocardiogram (ECG), and NIHSS. At 10 ± 2 days, we will perform assessments using the PRO-Stroke, repeated routine laboratory tests, ECG, and NIHSS. Biological samples will be collected at both baseline and 10 ± 2 days. The mRS, BI, and SS-QOL scores will be determined at 30 ± 3 days and 90 ± 7 days. Finally, vital signs and complications will be recorded at these four visits; on the other hand, AEs and SAEs will be recorded at any time during the trial.

### Data Collection and Management

Data collection and management will be performed in collaboration with clinical doctors and clinical research coordinators. All investigators in charge of patient recruitment, outcome assessment, data collection, and serum sample collection will receive pre-recruitment standardized training regarding this trial’s standard operating procedures. Investigators in all medical centers will make a reasonable effort to follow-up with the patient throughout the study period. Information obtained from patients will be recorded in the investigative case form by investigators; subsequently, the clinical research coordinator will perform data entry into electronic case report forms using a unique login ID. All patient-related information will be stored in locked file cabinets with limited access at medical centers. All serum samples, reports, data collection, and administrative forms will be only identified using a coded ID number to maintain participant confidentiality.

### Quality Control and Data Monitoring

The Steering Committee will be responsible for the scientific content of the protocol, overseeing the study operations, supervising the intra-study data sharing process, and preparing the primary manuscript and other publications arising from the EXPECT trial. Two contract research organizations will regularly perform data monitoring and data quality control. Data analysis will be completed by a third-party statistical unit (Tianjin University of Traditional Chinese Medicine).

### Adverse Events Management

All AEs will be evaluated for their association with investigational medicine; subsequently, they will be treated, recorded, and followed-up until recovery or stabilization. The investigators will report any SAE to the ethics committee, contract research organization, principal investigator, and China Food and Drug Administration.

### Sample Size Calculations

Based on a previous study that reported a mean decrease in the NIHSS score of 3 and 2.1 in the PNS and control groups, respectively, and a standard deviation of 2.5 (Wang, 2009), this trial will require 480 patients with a power of 90%, two-sided α of 0.05, and a 20% dropout rate to test the hypothesis that PNS is superior to placebo in decreasing the NIHSS score at 10 post-randomization days in patients with AIS.

### Statistical Analysis Plan

All randomized patients who receive at least one treatment dose and have safety outcome data will be included in the safety set. All patients in the safety set will be included in the full analysis set if efficacy outcome data are available. All patients in the full analysis set who are deemed to have no major protocol violations will be included in the per-protocol set. Efficacy and safety analyses will be performed according to the intention-to-treat principle. Additionally, per-protocol data analysis will be conducted as a reference. The last observation carried forward approach will be used to impute missing data of primary outcome. Regarding the primary outcome variable, between-group comparisons of the change in NIHSS score will be performed using Student’s *t*-test or Mann-Whitney *U* test, as appropriate. Regarding secondary outcome variables, between-group comparisons of the proportion of patients with an NIHSS score of 0–1, mRS grade ≤1, and BI score ≥90 will be performed using the chi-square test or Fisher exact test. Further between-group comparisons of the PRO-Stroke and SS-QOL score, as well as changes in biological indexes, will be compared using the *t*-test or Mann-Whitney *U* test. Moreover, between-group comparisons of the incidence of AEs will be compared using the chi-square or Fisher exact test. The effect of missing data on the results will be assessed through sensitivity analysis. All statistics will be 2-sided and statistical significance will be set at *p* < 0.05. Statistical analyses will be performed using SAS software version 9.4 (SAS Institute Inc.).

### Subgroup Analyses

Subgroup analyses for the primary outcome will be performed according to the following baseline characteristics: age (> 65 years vs. ≤ 65 years); gender (female vs. male); symptom onset to randomization time (≤24 h vs. > 24 h); disease history of hypertension, diabetes mellitus, coronary heart disease, stroke, and hypercholesterolemia; smoking history; Trial of Org 10,172 in Acute Stroke Treatment classification; main arterial stenosis; and stroke severity based on the NIHSS score.

## Discussion

Given that only a minority of patients with non-minor AIS could benefit from revascularization treatment, there remains a need for safe pharmacological neuroprotection against brain tissue injury in AIS treatment. Previous unsuccessful translational research on neuroprotective agents with unimodal targets has indicated the need for a single medicine blocking different key AIS-related mechanisms based on the complex pathophysiological cascade events of AIS (Rogalewski et al., 2006). Preclinical studies have reported the positive effects of PNS in alleviating inflammation injuries, anti-oxidation, and anti-apoptosis (Wan et al., 2009; Chen et al., 2011; Wang et al., 2011; Chen et al., 2015; Huang et al., 2015; Shi et al., 2017; Fu et al., 2018; Meng et al., 2019; Wu et al., 2019a; Zhang et al., 2019). Notably, exploratory studies using neuroimaging examination as the surrogate endpoint have demonstrated the therapeutic effect of PNS (Gui et al., 2013; Ren et al., 2018). However, the precise effect of PNS on patients with AIS should be further assessed using clinical endpoints.

As one of the main considerations in a trial design, the selection of an appropriate primary outcome is largely dependent on the disease and should reflect the treatment effect and expected mechanism. In this EXPECT trial, the post-intervention neurological improvement according to the NIHSS score is the primary clinical endpoint. The short time-span between treatment and NIHSS assessment requires relatively less effort to trace patients meanwhile reducing the risk of loss to follow-up. Researchers performed a causal mediation model using combined data from the MR CLEAN trial and IMS III trial and found that the change of NIHSS score reflected the treatment effect and lay on the causal pathway between treatment and long-term mRS categories. The results suggested that the NIHSS measures both neurological deficits and functional outcomes and that it could act as an alternative primary outcome for AIS treatment trials (Chalos et al., 2020).

To our knowledge, the EXPECT trial is the first multicenter, randomized, double-blind, placebo-controlled trial to assess the efficacy and safety of PNS in patients with AIS in the reperfusion era. This study is limited in terms of the lack of imaging assessment in follow-up procedures as unfavorable outcome predictors due to inadequate funding. However, we will explore the therapeutic mechanism of PNS therapy in the alleviation of cerebral ischemia-induced inflammatory damage. Moreover, the results will be valuable to interpret the efficacy of PNS. In summary, the EXPECT trial will provide critical evidence for PNS therapy for the vast majority of patients with AIS who are ineligible or have missed the opportunity for revascularization treatment.

## Abbreviations

PNS, Panax Notoginseng Saponins; NIHSS, National Institute of Health Stroke Scale; PRO-Stroke, Patient-Reported Outcomes scale for Stroke; mRS, modified Rankin Scale; BI, Barthel Index; SS-QOL, Stroke-Specific Quality of Life; ICAM-1, intercellular adhesion molecule-1; IL-6, interleukin-6; TNF-α, tumor necrosis factor-α; MMP-9, matrix metalloproteinase-9.
