# Supplementary material for: Efficacy and Safety of Panax Notoginseng Saponins (Xueshuantong) in Patients With Acute Ischemic Stroke (EXPECT) Trial: Rationale and Design
Source: Front Pharmacol. 2021 Apr 22;12:648921. doi: 10.3389/fphar.2021.648921 (PMC8101545; doi:10.3389/fphar.2021.648921)
Supplement: Supplementary file 2 [file datasheet1.pdf]

Supplementary materials

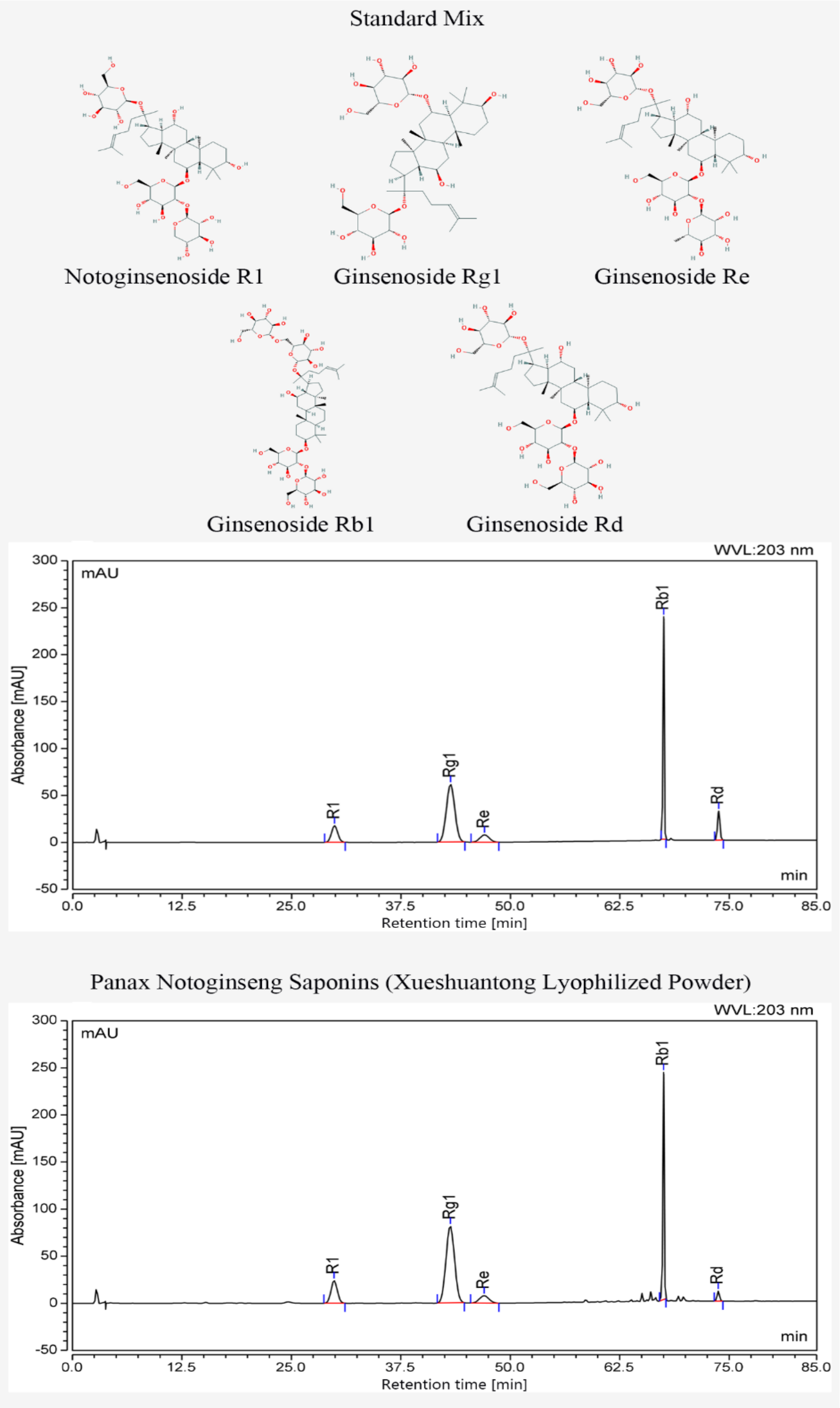

High performance liquid chromatograph (HPLC) of standard mix and Panax notoginseng saponins (Xueshuantong lyophilized powder). The HPLC separation was carried out using Ultimate 3000 high performance liquid chromatograph (Thermo Fisher Scientific), equipped with a Inertsil ODS-3 column (4.6mm × 250 mm, 5 µm). The mobile phase A was acetonitrile, and the mobile phase B was water. The elution gradient was shown as follows: (1) 0~45min, 20% A, 80% B; (2) 45~65min, 20→34% A, 80→66% B; (3) 65~85min, 34% A, 66% B; (4) 85~86min, 34→90% A, 66→10% B; (5) 86~96min, 90% A, 10% B; (6) 96~97min, 20% A, 80% B. The column temperature was set at 25 oC, and the flow rate was set at 1 mL / min.

R1, Notoginsenoside R1. Rg1, Ginsenoside Rg1. Re, Ginsenoside Re. Rb1, Ginsenoside Rb1. Rd, Ginsenoside Rd.

Table. Qualitative analysis of chemicals in Panax notoginseng saponins (Xueshuantong lyophilized powder)

Chemicals (PubChem Identifier)	Formula	Retention time (min)	Peak area (mAU*min)	Peak height (mAU)	Resolution (USP)	Relative peak area (%)
Notoginsenoside R1	$C_{47}H_{80}O_{18}$	34.483	24.659	25.97	9.50	11.74
Ginsenoside Rg1	$C_{42}H_{72}O_{14}$	50.000	113.499	102.05	2.09	54.06
Ginsenoside Re	$C_{48}H_{82}O_{18}$	52.795	12.398	21.99	27.03	5.90
Ginsenoside Rb1	$C_{54}H_{92}O_{23}$	70.455	55.247	211.31	18.68	26.31
Ginsenoside Rd	$C_{48}H_{82}O_{19}$	82.758	4.162	7.35	n. a.	1.98
